# Fine epitope signature of antibody neutralization breadth at the HIV-1 envelope CD4-binding site

**DOI:** 10.1172/jci.insight.97018

**Published:** 2018-03-08

**Authors:** Hao D. Cheng, Sebastian K. Grimm, Morgan S.A. Gilman, Luc Christian Gwom, Devin Sok, Christopher Sundling, Gina Donofrio, Gunilla B. Karlsson Hedestam, Mattia Bonsignori, Barton F. Haynes, Timothy P. Lahey, Isaac Maro, C. Fordham von Reyn, Miroslaw K. Gorny, Susan Zolla-Pazner, Bruce D. Walker, Galit Alter, Dennis R. Burton, Merlin L. Robb, Shelly J. Krebs, Michael S. Seaman, Chris Bailey-Kellogg, Margaret E. Ackerman

**Affiliations:** 1 Thayer School of Engineering and; 2 Molecular and Cellular Biology Program, Dartmouth College, Hanover, New Hampshire, USA.; 3 Faculty of Medicine and Biomedical Sciences, University of Yaoundé 1, Yaoundé, Cameroon.; 4 Department of Immunology and Microbiology, The Scripps Research Institute, La Jolla, California, USA.; 5 Unit of Infectious Diseases, Department of Medicine, Solna, Karolinska Institute, Stockholm, Sweden.; 6 US Military HIV Research Program, Walter Reed Army Institute of Research, Silver Spring, Maryland, USA; Henry M. Jackson Foundation for the Advancement of Military Medicine, Bethesda, Maryland, USA.; 7 Department of Microbiology, Tumor and Cell Biology, Karolinska Institute, Stockholm, Sweden.; 8 Duke Human Vaccine Institute, Durham, North Carolina, USA.; 9 Department of Medicine, Geisel School of Medicine at Dartmouth, Hanover, New Hampshire, USA.; 10 DarDar Health Programs, Dar es salaam, Tanzania.; 11 Tokyo Medical and Dental University, Tokyo, Japan.; 12 Department of Pathology, NYU School of Medicine, New York, New York, USA.; 13 Departments of Medicine and Microbiology, Icahn School of Medicine at Mount Sinai, New York, New York, USA.; 14 Ragon Institute of MGH, MIT, and Harvard University, Cambridge, Massachusetts, USA.; 15 Howard Hughes Medical Institute, Chevy Chase, Maryland, USA.; 16 Beth Israel Deaconess Medical Center, Boston, Massachusetts, USA.; 17 Department of Computer Science, Dartmouth College, Hanover, New Hampshire, USA.

**Keywords:** AIDS/HIV, Vaccines, AIDS vaccine, Adaptive immunity, Immunoglobulins

## Abstract

Major advances in donor identification, antigen probe design, and experimental methods to clone pathogen-specific antibodies have led to an exponential growth in the number of newly characterized broadly neutralizing antibodies (bnAbs) that recognize the HIV-1 envelope glycoprotein. Characterization of these bnAbs has defined new epitopes and novel modes of recognition that can result in potent neutralization of HIV-1. However, the translation of envelope recognition profiles in biophysical assays into an understanding of in vivo activity has lagged behind, and identification of subjects and mAbs with potent antiviral activity has remained reliant on empirical evaluation of neutralization potency and breadth. To begin to address this discrepancy between recombinant protein recognition and virus neutralization, we studied the fine epitope specificity of a panel of CD4-binding site (CD4bs) antibodies to define the molecular recognition features of functionally potent humoral responses targeting the HIV-1 envelope site bound by CD4. Whereas previous studies have used neutralization data and machine-learning methods to provide epitope maps, here, this approach was reversed, demonstrating that simple binding assays of fine epitope specificity can prospectively identify broadly neutralizing CD4bs–specific mAbs. Building on this result, we show that epitope mapping and prediction of neutralization breadth can also be accomplished in the assessment of polyclonal serum responses. Thus, this study identifies a set of CD4bs bnAb signature amino acid residues and demonstrates that sensitivity to mutations at signature positions is sufficient to predict neutralization breadth of polyclonal sera with a high degree of accuracy across cohorts and across clades.

## Abstract 2

Antibody fine epitope specificity can serve as a powerful tool in neutralization prediction for both mAbs and polyclonal sera.

## Introduction

Advancements in donor identification, experimental methods, and the generation of better reagents for cloning pathogen-specific antibodies have been realized in the past decade (1–3). These innovations have led to rapid growth in the number of broadly neutralizing antibodies (bnAbs) identified against clinically relevant pathogens, including HIV-1 (4–11). However, the ability to unambiguously translate binding to recombinant protein in biophysical assays into an understanding of in vivo activity against HIV-1 has lagged behind. In particular, there is a lack of concordance between antibody recognition of recombinant antigen in biophysical assays and broad antiviral neutralization activity in cell- and pseudovirus-based functional assays in the setting of humoral responses to HIV-1 infection and vaccination. Consequently, identification of subjects and mAbs with potent antiviral activity has generally remained reliant on empirical evaluation of neutralization potency and breadth. Such distinctions suggest that further work aimed at refining the properties of the antigen-derived protein probes used to characterize binding patterns of humoral responses may contribute to more efficient and effective identification of both protective mAbs and polyclonal antibody (pAb) responses.

HIV-1 bnAbs are of high interest due to their ability to prevent infection in animal models (12–16) and influence viral loads and host responses in humans (17–21). Numerous such bnAbs have been isolated and mapped to different regions of the HIV-1 spike, including the V1/V2 loop region, the V3 loop region, the membrane proximal extracellular region, the gp120-gp41 interface, and the CD4 receptor–binding site (CD4bs) (22–27). Among these, the CD4bs is of particular interest as a target for both mAb therapy and prophylaxis as well as for vaccine design. Despite the high sequence variability of the virus envelope, the CD4bs is functionally conserved and less masked by the glycan shield, and numerous potent bnAbs have been mapped to this region. Intriguingly, while many infected and vaccinated subjects raise CD4bs-specific antibodies, only a subset is broadly or potently neutralizing (28). Among these, convergent binding modes and common maturation pathways have been observed (29–31). Strikingly, differences in the angle of approach of broad or nonneutralizing CD4bs antibodies have been noted, as have differences in the fine epitope footprints (32, 33). Taken together, these findings suggest an association between CD4bs antibody fine epitopes and the ability to broadly and potently neutralize HIV-1.

Here, we undertook a study to evaluate the fine epitope specificity of a panel of CD4bs mAbs using a designed library of YU2 gp120 core amino acid point mutants (34), and, in doing so, we define the molecular recognition features of functionally potent humoral responses to the HIV-1 envelope CD4bs. Whereas previous studies have used neutralization data to delineate antibody epitopes (35–40), here that process is reversed to allow prediction of neutralization potency from epitope-mapping experiments. Employing both computational models and experimentation, we demonstrate that biophysical definition of antibody fine epitope specificity can contribute to prospective identification of broadly neutralizing CD4bs–specific mAbs. Building on this result, we further show that epitope mapping and predictions of neutralization breadth can be accomplished in the setting of polyclonal serum responses.

## Results

### Fine CD4bs epitope specificity predicts neutralization breadth.

**Figure 1:**
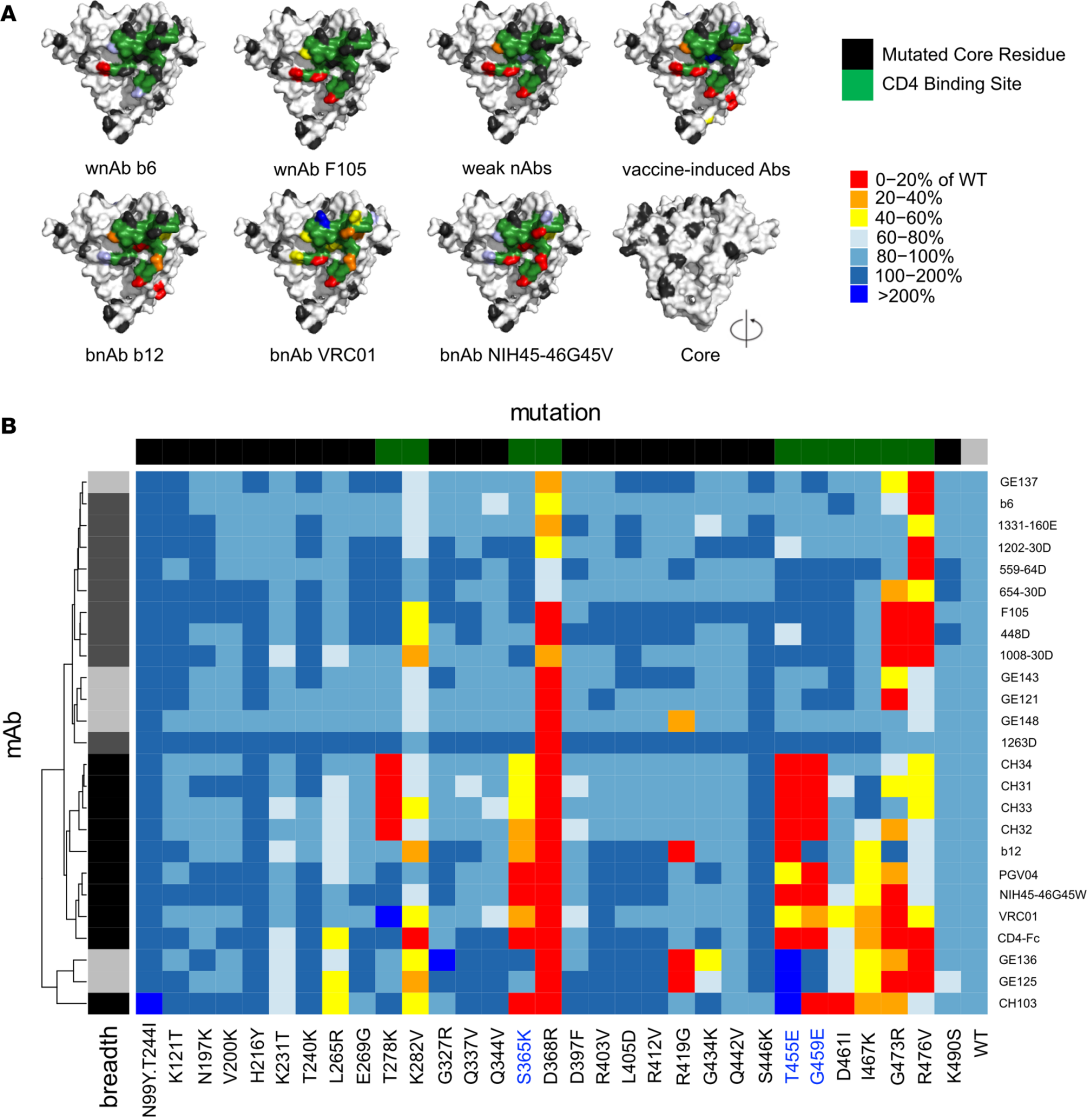
Epitope mapping of CD4bs antibodies. A panel of gp120 core point mutants was used to epitope map CD4bs antibodies (*n* = 25). Representative mAbs from 4 groups, including weakly neutralizing (wnAbs), vaccine-induced, and broadly neutralizing (bnAb) antibodies were evaluated. (**A**) The sensitivity of various mAbs to mutation of core residues is plotted on a structural representation. The CD4bs is colored green, and tolerated point mutations are colored black, while substitutions driving reduced (<80%; light blue to red) or strongly enhanced (>160%; blue) binding to the core relative to WT are indicated. (**B**) Heatmap representation of the epitope-mapping results observed for the set of CD4bs mAbs. Hierarchical clustering identifies major subgroups of CD4bs mAbs that are associated with neutralization breadth and potency. The color bar at top indicates the class of core: core variants with substitutions made in CD4bs residues are indicated in green, core variants with substitutions made in other sites on the core indicated are indicated in black, and the unmutated WT core are indicated in gray. The color bar at the left indicates the mAb class, with bnAbs indicated in black and non-bnAbs indicated in shades of gray (vaccine-induced antibodies in light gray, infection-induced antibodies in dark gray).

To define antibody epitopes, we mapped a set of approximately 30 single amino acid substitution variants of yeast-displayed YU2 gp120 core (34) via flow cytometry for disruption of binding across a panel of CD4bs antibodies. We evaluated 9 antibodies with narrow and/or weak neutralizing activity (wnAbs) (41), including b6 and F105; 6 vaccine-induced non-bnAbs from macaques (viAbs) (42); 9 antibodies with more broad and potent neutralization activity (bnAbs; b12, VRC01, PGV04, NIH45-46 G45W, CH31-CH34, and CH103); and the natural ligand CD4. This set of CD4bs mAbs, as well as CD4, generally shared a common pattern of sensitivity toward 4 residues, K282, D368, G473, and R476, all located along the CD4bs ridge facing the inner core domain (representative examples, Figure 1A). However, hierarchical clustering demonstrated that the epitope maps of CD4bs bnAbs could be differentiated from the non–broadly neutralizing (non-bnAb) CD4bs viAbs and wnAbs (Figure 1B), indicating a common difference in CD4bs recognition that was associated with neutralization breadth. The group of clustered bnAbs was distinguished by sensitivity to point mutations deeper within the CD4bs (Figure 1A), associated with S365K, T455E, and G459E substitutions (Figure 1B). Thus, both visual inspection and hierarchical clustering indicated that epitope maps could be used to discriminate neutralization breadth among CD4bs mAbs.

### Validation of bnAb epitope signatures to predict CD4bs antibody neutralization breadth.

**Figure 2:**
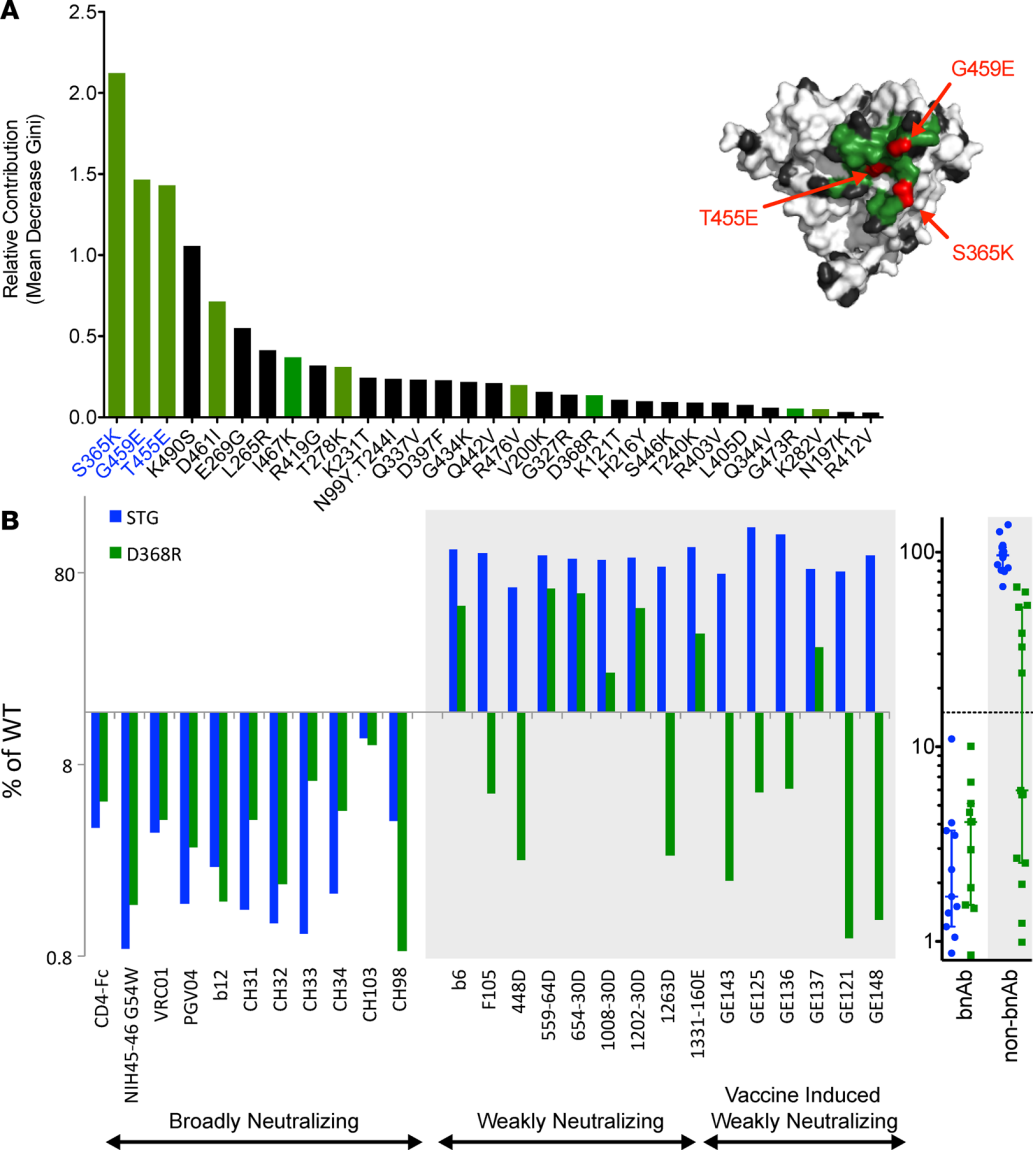
Classification of CD4bs mAb neutralization breadth. (**A**) A random forest approach was used to classify mAb neutralization breadth using epitope maps. The relative importance of each point mutant to the classification models is presented in decreasing order, as ranked based on mean decrease in Gini index. CD4bs residues are colored in green, and other core residues are colored black. A structural model of the core, denoting the locations of the top 3 positions (S365, T455, G459) utilized by the classifier in red, is shown. (**B**) Benchmarking against D368R. The residues most important to the classifier were mutated to generate a triple mutant probe (STG). The binding of each CD4bs mAb (*n* = 26) relative to the WT gp120 core is presented for STG and the CD4bs probe D368R across individual mAbs and when grouped according to neutralization breadth. Data are represented as median and interquartile range.

To further investigate this observation, a random forest model (43), which corrects for decision trees’ propensity to overfit data, was trained to classify the CD4bs mAbs by neutralization breadth (bnAb versus viAb and wnAbs) via the generation of an ensemble of decision trees, using epitope-mapping data as input. Perfect classification accuracy was achieved across the set of mAbs in the setting of leave-one-out cross-validation (Table 1). The relative importance of each mutant in the panel to predictively classifying neutralization breadth across the forest of decision trees identified positions S365, G459, and T455 (Figure 2A) as contributing the most toward classifier predictions. As might be expected, the 10 mutated CD4bs residues were generally ranked higher than mutations at other positions in the gp120 core. The widely used CD4bs-defining D386R mutation (44) ranked among the bottom half of variants, providing essentially no contribution to neutralization breadth classification, which is consistent with the sensitivity of all CD4bs mAbs tested to this mutation. Similar performance and contributing features were observed using elastic net classification as an alternative modeling approach, supporting the generalized ability of this epitope recognition signature to predict neutralization breadth. Finally, permutation tests, in which models were learned from data when the neutralization class labels had been scrambled, further established model robustness.

Based on these models, a triply mutated variant of the gp120 core, denoted as STG and consisting of S365K, T455L, and G459E substitutions, was generated by site-directed mutagenesis in order to evaluate bnAb signature substitutions in combination. The sensitivity of each CD4bs mAb to the substitutions present in the STG mutant and to the D368R substitution was evaluated relative to WT core. Whereas all CD4bs mAbs were sensitive to substitution at the D368 position, as evidenced by an average 75% decrease in binding signal for the D368R variant relative to WT core, only bnAb recognition was dramatically affected by the STG triple mutation (Figure 2B). While the D368R mutation did tend to be more disruptive to bnAbs than non-bnAbs, recognition by more than half of the non-bnAbs was reduced by a factor equivalent to that of the bnAbs. In contrast, antibody binding to the STG mutant was generally unaffected for non-bnAbs, maintaining an average of 97% of WT signal, whereas it was dramatically decreased among bnAbs, averaging 3% of WT signal (*P* = 3.1 × 10^–12^ by 2-tailed Mann-Whitney test). Thus, consistent with the hierarchical clustering and random forest classification results, in which bnAbs and non-bnAbs could be discriminated statistically with the mutant panel using models weighted heavily on the 3-position signature, evaluation of the STG triple mutant confirmed robust discrimination capacity experimentally. A limited set of other substitutions was made at these positions and demonstrated similar binding profiles, indicating that bnAb sensitivity was likely more position rather than substitution specific.

### Epitope mapping of polyclonal sera.

**Figure 3:**
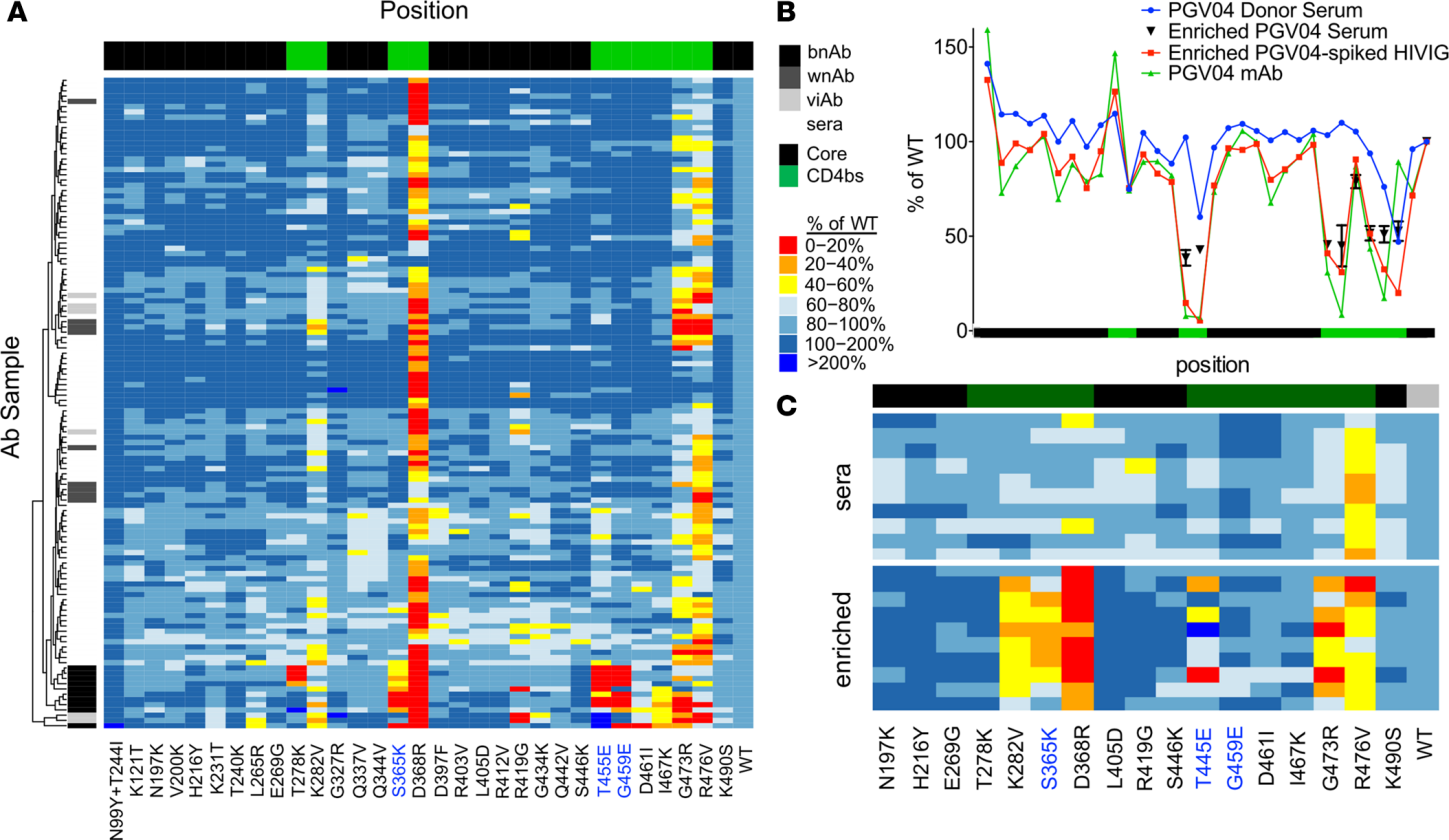
Polyclonal sera epitope-mapping results. (**A**) Heatmap comparing epitope-mapping data from polyclonal sera from Boston area (Ragon Institute) HIV-infected subjects positive for YU2 gp120 core-specific antibodies clustered together with the mAb epitope maps. The vertical color bar indicates sample type: pAb samples are shown in white (*n* = 99), bnAbs in black (*n* = 10), vaccine-induced weakly neutralizing antibodies (viAbs, *n* = 6) in light gray, and infection-induced weakly neutralizing antibodies (wnAbs, *n* = 9) in dark gray. (**B**) Serum from a donor with a known bnAb (PGV04) was evaluated for binding to the epitope-mapping panel. The ability of the STG mutant to deplete core-specific antibodies without broad neutralization and to facilitate identification of the presence of CD4bs antibodies with broad neutralization was determined for the PGV04 donor serum and for PGV04-spiked HIVIG. Error bars, shown only for the enriched PGV04 serum, indicate SD observed between duplicate measurements. (**C**) Heatmap of polyclonal sera samples from a Tanzanian cohort (*n* = 10) before and after enrichment of potential bnAbs using the STG mutant-based depletion. Horizontal color bars indicate the class of core: core variants with substitutions made in CD4bs residues are indicated in green, core variants with substitutions made in other sites on the core are indicated in black.

Based on excellent discrimination among mAbs, we next evaluated whether the mutant panel could be used to epitope map pAb samples derived from serum. A set of 121 samples from chronically infected subjects from Boston area cohorts was screened for binding to yeast-displayed YU2 core, and the 99 samples with significant YU2 core reactivity were further evaluated across the entire mutant panel (Figure 3A). While essentially all serum samples with core-specific antibodies were highly sensitive to the D368R mutation and moderately sensitive to substitutions at G473 and R476, they did not bear appreciable sensitivity to the neutralization breadth signature residues. When clustered alongside the maps from the CD4bs mAb panel (Figure 3A), the pAb-binding signatures were clearly more similar to the wnAbs and viAbs than the group of bnAbs, which formed their own branch on the dendrogram. Indeed, because neutralizing responses were expected among chronically infected subjects (45), the lack of any CD4bs bnAb-like epitope signatures suggested that either the mAb-derived signature was not a general feature of pAb responses or that more prevalent responses toward the lower CD4bs ridge might impede detection of less common antibodies able to interact with the CD4bs neutralization signature residues.

To investigate the hypothesis that CD4bs non-bnAbs may mask the detection of CD4bs bnAbs, serum from the CD4bs bnAb PGV04 donor was epitope mapped (Figure 3B). Indeed, despite the presence of a bnAb, serum from the PGV04 donor failed to recapitulate characteristics of the PGV04 mAb and appeared similar to the other serum samples evaluated: sensitivity to the bnAb signature residues was not observed, but reduced binding to D368R and R476V mutants was noted. Similar results were observed when known concentrations of the PGV04 mAb were spiked into HIV immune globulin (HIVIG), confirming the hypothesis that nonneutralizing core-specific antibodies can impede detection of bnAbs in mapping approaches that rely on the gp120 core. This observation suggested that depleting pAb samples of non-bnAbs using the STG mutant might result in the enrichment of CD4bs bnAbs. This possibility was experimentally tested by passing PGV04-spiked HIVIG through a spin column containing a bed of STG-displaying yeast and epitope mapping the column flow through. Similar serum adsorption steps have contributed to previous gp120 epitope-mapping studies (46). This enrichment step enabled identification of sensitivity toward substitution of critical residues recognized by the PGV04 mAb, demonstrating that this flow-through fraction was enriched for antibodies with the PGV04 phenotype. Similarly, though insufficient PGV04 donor serum was available to evaluate the STG-enriched fraction against the whole epitope panel, a subset of the panel was analyzed for binding to the enriched fraction. Sensitivity to S365K, T455E, G459E, I467K, and G473R, key features of the PGV04 mAb that were not observed in whole PGV04 donor serum, was observed following enrichment (Figure 3B).

**Figure 4:**
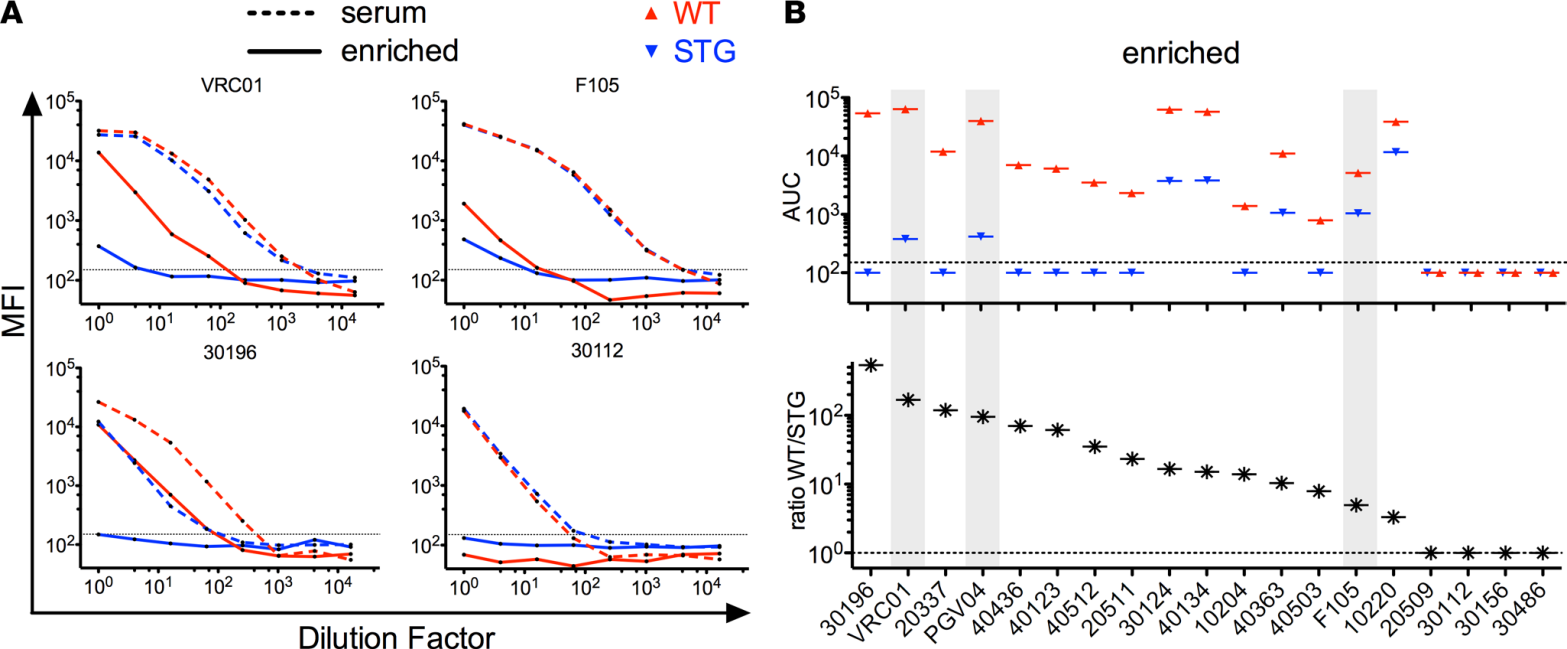
Inferring the presence of CD4bs bnAbs via STG-based enrichment and serum profiling. A set of 16 samples from the RV217 HIV cohort were titrated for binding to WT (red) and STG (blue) core before and following enrichment of CD4bs bnAbs via STG-based depletion to enable inference of the presence or absence of CD4bs bnAbs. VRC01, PGV04, or F105 were spiked into HIV immune globulin (HIVIG) as positive and negative controls. (**A**) Titration curves of VRC01-spiked and F105-spiked HIVIG (top), and two RV217 subjects (bottom) before (broken line) and after (solid line) enrichment. The dotted line indicates the signal baseline used for AUC calculations. (**B**) The AUC for all sample titrations against WT and STG cores after enrichment was calculated and samples were plotted by rank in the ratio of the AUC for WT relative to STG. The mAb-spiked samples are highlighted in gray. Samples with no measurable binding were assigned a value of 100.

Inspired by the observation that STG-based enrichment could unmask the presence of a CD4bs bnAb, we extended this enrichment process across a set of 10 serum samples from a Tanzanian cohort. In a pilot experiment, enriched sera (the flow-through fraction that results when the pool is depleted by passage through a column of STG yeast cells) were analyzed for differential binding to a subset of the mutant panel, and the majority of these demonstrated differences in epitope-mapping results before and after enrichment (Figure 3C). The difference between before and after enrichment maps that was most apparent was increased sensitivity to K282V, S365K, and G473R substitutions. Limited sensitivity to T455E was noted, and none of these samples demonstrated sensitivity to G459E. When the neutralization activity of this limited panel of serum samples was determined, only weak and narrow responses were noted, consistent with the lack of responses to the substitutions characteristic of CD4bs bnAbs. While the Tanzanian serum samples were relatively insensitive to T455E and G459E, and were also nonneutralizing, suggesting the utility of this mapping approach, we wished to more meaningfully explore the utility of this epitope signature by applying it to samples with known neutralization profiles. To this end, we applied the enrichment process to a set of 16 serum samples from the RV217 cohort that were known to be broadly neutralizing but for which epitope mapping based on neutralization data (40) suggested a wide range of epitope specificities. As positive and negative controls, mock samples consisting of VRC01, PGV04, or F105 mAbs spiked into HIVIG (5% by mass) were generated and subjected to the same enrichment process in parallel. Diverse titration patterns were observed across the set of 16 sera, with some subjects exhibiting depletion patterns similar to that of VRC01-spiked HIVIG and others more similar to the F105-spiked sample (Figure 4A). To quantitate these differences, the area under the titration curve (AUC) was calculated, and all samples were ranked by the ratio of enriched WT-AUC over enriched STG-AUC (Figure 4B), as sera samples with a higher ratio would be predicted to be more likely to contain CD4bs bnAbs. Notably, among these samples, one subject demonstrated a binding profile superior to that observed for either the VRC01 or PGV04 spike-in samples, and a number of subjects exhibited profiles intermediate between these bnAbs and the F105-spiked sample. These results suggest the potential utility of the STG mutant in B cell cloning and bnAb isolation efforts as well as in assessing bnAb prevalence more precisely in heterogeneous populations.

**Figure 5:**
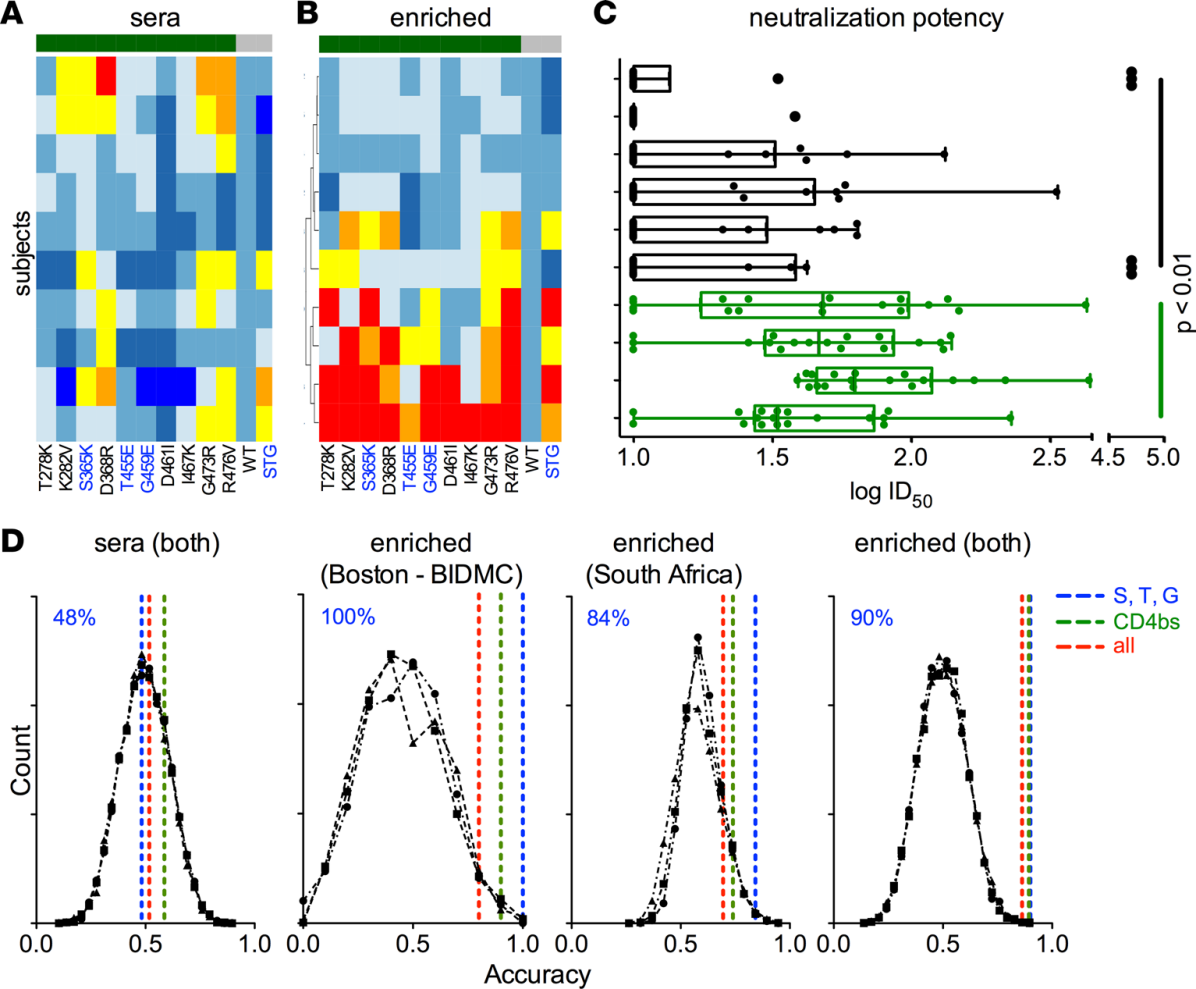
Prediction of polyclonal sera neutralization breadth across diverse subject cohorts. Heatmap of the relative binding of sera before (**A**) and after (**B**) STG-based enrichment for a set of 10 samples from a second Boston area (BIDMC) cohort against CD4bs mutant cores (green), the WT core, and the triple mutant STG core (both in gray). (**C**) Neutralization potency (ID_50_) of the 10 sera samples across a panel of 18 tier 2 virus strains (each dot represents 1 unique virus strain) with subjects aligned as in **A** and **B**. Interquartile ranges and Tukey whiskers are shown; mean ID_50_ values across the virus panel for each subject were compared between the STG sensitive (green) and insensitive (black) group, with significance defined by Mann-Whitney test. (**D**) A classifier was trained to predict the neutralization breadth of polyclonal samples from HIV-infected subjects in the Boston area (BIDMC) cohort (*n* = 10), a South African cohort (*n* = 19), or both, based on epitope-mapping input of sera or the STG-enriched IgG fraction. Dashed lines represent classifier performance when the complete mutant panel (red), CD4bs residues only (green), or the S, T, and G positions alone (blue) were used in model training. Black lines represent classifier performance when permuted neutralization class assignments were predicted using the whole panel or CD4bs and S, T, and G mutant subsets. Classification accuracy of models learned from considering S, T, and G positions only are noted at the top left of each panel in blue.

To link single substitution-level epitope maps of enriched pAb fractions to pAb neutralization activity, samples with known neutralization potency from a second, independent Boston area cohort were evaluated. Recognition profiles before (Figure 5A) and after (Figure 5B) CD4bs bnAb enrichment using the STG mutant were defined for a set of 10 serum samples. Clustering subjects based on the maps following enrichment identified a subset of individuals with broad reactivity against the CD4bs, including sensitivities to bnAb signature residues that were only apparent following enrichment.

The neutralization potency of these samples across a panel of 18 tier 2 viruses was tested (Figure 5C). Those samples that clustered together as being most strongly disrupted by CD4bs substitutions following STG-based enrichment exhibited potentiated neutralization capacity (as defined by the mean ID_50_ across the virus panel for each subject) relative to the cluster of samples that were relatively insensitive to CD4bs substitutions (*P* < 0.01 by 2-tailed Mann-Whitney test).

### Prediction of serum neutralization breadth using the bnAb epitope signature.

The presence of the post hoc association described suggested that epitope maps following STG-based enrichment might support predictive models of neutralization breadth in polyclonal samples. Therefore, we again utilized the random forest machine-learning method to train a classifier to distinguish neutralization breadth. We evaluated two cohorts, the 10 Boston area subjects described above and an additional cohort of 19 South African subjects to investigate whether this approach could be generalized to clade C infection. Predictions of neutralization breadth class (high versus low) were made using all 31 mutations, the 10 CD4bs residues, or the 3 bnAb signature positions S, T, and G (Figure 5D and Table 2). Because the neutralization data for pAbs were collected from a limited panel of pseudoviruses and for a limited number of subjects, neutralization potency was divided into two groups: broadly neutralizing and non–broadly neutralizing, rather than across a continuum of activities. As with any such classification exercise, results of this analysis are expected to be somewhat dependent on the specific boundary used to define the two groups.

Permutation tests were conducted to evaluate the classification error distribution when neutralization class labels were scrambled. These tests established a baseline performance expectation that served as a negative control. When native serum maps were used, the performance of predictive models was essentially random, demonstrating accuracies of approximately 0.5 in defining the two classes. This performance was indistinguishable from the error distribution when permuted neutralization class assignments were predicted. In contrast, when the epitope-mapping data following the STG-based enrichment were used, all residue sets demonstrated accuracies that were significantly better than predicted by chance alone (Table 2). Strikingly, the best predictive performance was observed when only the 3 bnAb signature positions were utilized (Figure 5D), demonstrating that this motif was sufficient to effectively classify the breadth and potency of neutralization activity present. When these signature positions were excluded from consideration, model performance was significantly degraded, demonstrating that beyond being sufficient, these positions were also necessary for best results. Furthermore, both cohorts, which represent subjects infected primarily with either clade B or clade C viruses, demonstrated similar performance and reliance on the S, T, and G positions, suggesting that predictive models were neither cohort nor clade specific, but were generalizable. Indeed, when cohorts were combined, similar performance accuracy was observed, suggesting that similar decision trees effectively classified subjects from both cohorts. Collectively, these results suggest that STG-based enrichment was necessary, and that this enrichment approach was sufficient to enable prediction of neutralization breadth of polyclonal sera across cohorts and across clades with a high degree of accuracy.

## Discussion

Considerable effort has been expended to identify and characterize antibodies with the ability to neutralize diverse HIV-1 variants. Such bnAbs are not only potentially useful in prevention and treatment, but they also offer a model for vaccine design in that they encapsulate the natural developmental history and illuminate features of potentially protective humoral immune responses. Since the first identification of HIV-1 bnAbs in the early 1990s, almost 100 bnAbs have been described (47), and a new wave of clinical studies evaluating their utility has been initiated. In particular, a number of CD4bs bnAbs are currently being investigated for antiviral therapy and prophylaxis (17–21). Similarly, diverse vaccine design strategies have focused on the CD4bs, involving approaches that have matured from obscuring other surface residues (48), to minimizing the presence of other epitopes (49), to grafting the CD4bs onto alternative scaffolds (50), to designing modified CD4bs epitopes favoring interactions with specific germline B cell receptors (51), to selecting immunogens based on B cell and viral coevolution (52). These and other examples highlight the importance of the CD4bs in the design of both HIV-1 vaccines and antiviral antibody drugs.

The promising new work devoted to these efforts provides reason to be optimistic that eliciting CD4bs bnAbs by vaccination is possible and suggests that effective characterization of the antibody responses being raised by novel vaccine candidates may serve a critical role in vaccine research and development. While other antibody phenotypes, such as loop length and CDR composition, have been used as surrogates to suggest progress toward neutralization breadth, such proxies are unlikely to be either strongly or universally associated with neutralization. Similarly, given the insufficiency of CD4 or antibody competition experiments to fully reflect neutralization potency, we expect that more sophisticated epitope-mapping probes and strategies, such as that defined here, may be central to ongoing efforts to profile mAbs and pAb responses (53, 54). These methods may help in particular by providing more fine-grained information about modes of recognition that may then better inform the design of future immunogens or immunization sequencing regimens.

Here, we show that prior knowledge as to function-phenotype linkages can be used to develop robustly predictive models of the neutralization phenotype observed for samples based on antigen recognition profiles. While inferences of neutralization potency made from patterns of binding recognition learned from examples could likewise be made from epitope information gained from other experimental methods, such as cocrystallization, hydrogen-deuterium exchange mass spectrometry, and scanning and shotgun mutagenesis, the yeast display-based approach has considerable experimental advantages. Similarly, as appropriate sets of antibodies become available, defined antigen variants could be developed to distinguish neutralization breadth and potency at other epitopes of interest.

Whereas the current state of the art in pAb epitope mapping involves using experimental neutralization data to predict HIV-1 antibody epitopes (35–39), here the directionality of inference is reversed, and, to our best knowledge, this is the first time that polyclonal HIV-1 antibody epitope-mapping data were used to predict neutralization potency. While numerous laboratories have established high-throughput means to conduct neutralization assays, biophysical probes bear advantages in cost, ease, and safety (55). Significantly, distillation of a panel of single amino acid point mutants into a single, triply mutated variant enabled highly efficient experimental discrimination by simply evaluating binding to WT versus triple mutant core. Collectively, these data also suggest that B cell sorting of B cell receptors that prefer WT to the STG mutant could contribute to efforts to identify and clone CD4bs bnAbs.

However, generalization of this epitope-based approach to deduce neutralization has a number of limitations. First, it relies on the availability of antibodies with known activity profiles and thus has little relevance to settings in which such rich knowledge does not yet exist. Second, since it is built on prior knowledge, it is expected to have a limited ability to identify antibodies with novel recognition modes. If there are no examples in the class of antibodies used to train models and build a discriminatory epitope probe, the designed probe will presumably have no ability to recognize new antibodies that may exhibit the same functional phenotype but rely on a different biophysical one.

We also note that while the STG probe was effective in distinguishing neutralization potency among mAbs and for the cohorts (Beth Israel Deaconess Medical Center [BIDMC] and South Africa) in which neutralization data were available, additional data are needed to further validate these observations. Relevant to this concern, epitope maps (before and/or after enrichment) from other cohorts (Ragon Institute and Tanzania) differed from those observed among subjects for which neutralization data were available in several ways. For example, prior to enrichment, D368R was strongly disruptive in the majority of subjects from the Ragon Institute cohort; this phenotype was considerably less prevalent among the BIDMC (and Tanzanian) subjects. Additionally, after enrichment, the BIDMC cohort samples with broad neutralization potency exhibited multiple sites of substitution sensitivity, and this broad pattern of disruption across the CD4bs was distinct from that observed among Tanzanian subjects after enrichment. Such differences in both the before and after enrichment epitope maps among the samples from which neutralization data were available could be potentially explained by a number of factors, including time since infection, concomitant disease burdens, or differences in recognition of YU2 core associated with diversity in the infecting virus, among others. By extension, we cannot exclude that other factors such as these may influence the utility of this approach to predict neutralization activity.

Our results also suggest that, in general, the bulk of the antibody response in infected subjects that recognizes the gp120 core may not be relevant to virus neutralization. Indeed, it has long been known that much of the humoral response to HIV-1 infection is directed to “viral debris” (56). Our observation that non-bnAb responses can mask the presence of bnAbs has several important potential biological implications. First, this result is consistent with numerous previous studies reporting the inability of gp120-binding titers to strongly correlate with neutralization breadth and potency and with reports of limited success in isolating gp120-reactive antibodies with potent neutralizing activity (32, 57). That most core-specific antibody responses are apparently irrelevant to neutralization has implications as to the usefulness of gp120 or gp120 cores as vaccine immunogens. This observation also suggests that there could be competition between neutralizing core-specific antibodies and nonneutralizing core-specific antibodies and that binding of non-bnAbs might block bnAb activity. While we did not evaluate this possibility intensively, the inability of non-bnAbs to compete with nAbs has been long established (58). Further, in this study, neutralization enhancement was not observed when a subset of sera was evaluated for neutralization activity before and after STG-based depletion of non-bnAbs. Finally, and in contrast to our biophysical assays, the presence of PGV04 in the PGV04 donor serum was readily apparent in neutralization assays. Thus, these results indicate that, while non-bnAbs can mask biophysical detection of bnAbs, they likely do not mask bnAb neutralization activity either in vitro or in vivo. Finally, and perhaps surprisingly, we could observe good predictions based on assessment of core-binding profiles alone. This result suggests that CD4bs-specific antibodies may represent a dominant mode of neutralization or that they are correlated with the induction of bnAbs directed at other sites, at least among the samples evaluated here.

Overall, this study demonstrates that antibody fine epitope specificity can serve as a powerful tool in neutralization breadth prediction for both mAbs and polyclonal sera. We envision that the application of this method to additional panels of antibodies and antigen variants may bring more insights into future vaccine design against HIV-1 as well as other viruses and that the STG probe in particular may prove useful in ongoing efforts to evaluate humoral responses to candidate vaccines and to identification and cloning of novel bnAbs of the CD4bs class.

## Methods

### mAbs.

Six viAbs, including GE121, GE125, GE136, GE137, GE143, and GE148, were sourced from the Karolinska Institute (42). Seven narrowly and/or weakly neutralizing antibodies, including 448D, 559-64D, 654-30D, 1008-30D, 1202-30D, and 1263D, were sourced from the NYU School of Medicine (41). Five antibodies, including F105, b12, VRC01, and NIH45-46 G45W, were acquired from the NIH AIDS Reagent Program. Six antibodies, including CH31, CH32, CH33, CH34, CH98, and CH103, were provided by the Duke Human Vaccine Institute. The remaining CD4bs mAbs were provided by The Scripps Research Institute. The same set of mAbs was used in Figure 1, Figure 2, and Figure 3A.

### Clinical samples.

Serum samples from 176 HIV-infected subjects from cohorts, including chronically infected individuals from cohorts established by the Ragon Institute (*n* = 121); chronically infected individuals from the greater Boston area (*n* = 10) available from BIDMC; antiretroviral drug-naive HIV-1 clade C chronically infected individuals from the Southern African National Blood services (*n* = 19); individuals subsequently purified for immunoglobulin at the DarDar Study in Dar es Salaam, Tanzania (*n* = 10) (59); and the RV217 early capture HIV cohort study (*n* = 16) (ECHO) collected 1–3 years after infection and prior to ART (60) were evaluated. Cohorts were not controlled for variation in viral load, CD4 count, time from infection, ART therapy, age, sex, or other factors that may influence antibody responses. IgG present in sera from HIV-infected donors was purified using Pierce Melon Gel according the manufacturer’s instructions. A 5% Triton X-100 solution in PBS was used to bring each sample to 0.5% Triton X-100 before heating at 37°C for 1 hour to inactivate virus. Neutralization activity for the Boston and African sample sets was determined using the TZM.bl assay across an 18-virus panel as previously described (61). Neutralization class identity was determined by mean value of log-transformed ID_50_ across the panel of HIV-1 strains.

### Epitope mapping.

A panel of gp120 core mutants was induced and displayed on *Saccharomyces cerevisiae* strain EBY100 as previously described (34, 62). Amino acid substitutions S365K, T455L, and G459E were sequentially introduced using site-directed mutagenesis and confirmed by sequencing. Titrations were performed for each antibody sample in order to determine, first, whether a YU2 core-specific response was present and, second, to identify the dose-response inflection point. The concentration at which signal is half-maximal represents an optimal concentration for epitope mapping, at which binding to the core was most sensitive to concentration and good signal to noise resolution is observed. For both titrations and epitope maps, approximately 1 × 10^5^ yeasts displaying wild-type gp120 4G core were combined with 200 μl PBS + 0.1% BSA (PBSB) per well and centrifuged in 96-well plates at 3,200 *g* for 4 minutes. Supernatants were removed by aspiration, and cells were resuspended in 50 μl antibody solution containing titrations of mAbs or polyclonal sera and 1:400 mouse anti-HA tag antibody (Covance) and incubated with shaking for 1 hour at room temperature for mAbs or overnight at 4°C for human sera. Yeasts were washed twice with PBSB and stained for 20 minutes at room temperature with a 1:200 solution of fluorescent goat anti-mouse and anti-human/rhesus antibodies (1:200 each) to enable detection of surface displayed core and bound core-specific antibody, respectively. Plates were washed and resuspended in 200 μl PBSB, and data were acquired on a MACSQuant Analyzer (Miltenyi Biotec). The mean fluorescent intensity (MFI) of gp120 core-displayed yeast was determined by gating on the HA tag-positive cells, and normalized MFIs were determined by determining the ratio of test antibody signal relative to the level of core display (test antibody MFI/HA tag MFI). Assays were generally performed in singlicate, as variability in the epitope mapping assay was previously evaluated, and found to exhibit an inter-study coefficient of variation (%CV) of generally less than 10% and intra-study %CVs under 5% (53, 54).

### bnAb enrichment.

The STG-based bnAb enrichments were performed by depleting polyclonal pools of core-specific antibodies that recognized epitopes other than the 3-residue bnAb signature. Briefly, approximately 1.4 × 10^9^ yeasts were washed with PBS before being gently pelleted for 2 minutes at 500 *g* in a cellulose acetate filter column (Pierce). A small volume of PBS was added without disturbing the pellet, and a 30-μm polyethylene filter was placed on top of the pellet as a frit (Pierce). PBS was removed from above the frit, and pAb sample was added to the column and centrifuged until the solution passed through the column at 250 *g* (approximately 15 minutes). The flow through was collected and reapplied to the column and centrifugation repeated at 500 *g* for approximately 10 minutes. This process was repeated on a total of 3 yeast-based affinity columns to ensure complete depletion. Enriched pAbs were either evaluated by epitope mapping using the yeast-displayed core mutants as described above or evaluated by assessing binding to STG and WT-conjugated fluorescently coded magnetic beads. Soluble STG gp120 core and WT gp120 core protein were expressed by HEK cells (Invitrogen) and purified using standard Ni-NTA chromatography. STG gp120 and WT gp120 proteins were then conjugated to the magnetic beads via primary amines through the NHS-EDC chemistry, as previously described (63, 64). To detect pAb bound to STG/WT gp120, 30 μl enriched and unenriched pAbs were serially diluted in black, clear-bottom 384-well plates (Greiner Bio One) with a dilution factor of 4. For each specificity (STG and WT), 500 beads were added in a 20-μl volume to each well, followed by 1-hour incubation on a plate shaker at 1,050 rpm at room temperature. Plates were then washed with PBS with 1% BSA and 0.05% Tween and incubated in 50 μl anti-human IgG Fc-PE (Southern Biotech) at 650 ng/ml as a secondary antibody to detect bound pAbs for 30 minutes. Finally, plates were washed and beads were resuspended in 35 μl Luminex sheath fluid buffer. The net MFI was detected and reported by a FlexMap 3D (Luminex, Bio-Plex Manger 5.0, Bio-Rad). Area under the curve was calculated in GraphPad Prism.

### Data analysis and visualization.

Surface representations of YU2 gp120 core mutants and mapped epitopes were generated using PyMOL and were colored based on a modeled gp120 core structure as described previously (34). Heatmaps were plotted by the gplots package in R.3.1.0 with the heatmap.2 function and dendrograms were generated by hierarchical clustering (Euclidean distance). Classification models were built using the random forest decision tree package “randomForest” in R.3.1.0 (43). 20,000 decision trees were built for each binomial trainer per run. The relative importance of each epitope-mapping measurement to classification models was evaluated by the mean decrease in Gini index. Predictive accuracy was assessed by leave-one-out cross-validation. Model quality was also assessed by permutation tests, in which neutralization class identity was randomized with a fixed ratio between bnAbs and nnAbs and the classifier performance was evaluated over 1,000 different permutations. Classification models with accuracies greater than 2 SDs above than the permuted model mean were regarded as significant. As methodological alternatives, elastic net models were also built using the Glmnet package (65), with an elastic net mixing parameter at 0.4, and use of leave-one-out cross-validation to determine the value of the tuning parameter lambda, such that minimum cross-validated mis-classification error was observed. Similar classification performance and common contributing features were observed across random forest and elastic net methods. Results from random forest models were selected for presentation, as this method is robust to outliers, computationally efficient, and resistant to overfitting (66).

### Statistics.

Subjects were clustered into groups defined by CD4bs epitope-mapping data, and the median neutralization ID_50_ values observed for individuals from each group were compared. A *P* value of less than 0.01 was considered significant. Classification models were considered to perform significantly better than expected at random if their accuracies were greater than 2 SDs above the mean accuracy observed from models learned from permuted data. Comparison of neutralization ID_50_ values was conducted by Mann-Whitney test in R 3.3.1 with the wilcox.test function.

### Study approval.

All subjects were adults, and they provided written informed consent. The Dartmouth College Committee for the Protection of Human Subjects approved the study.

## Author contributions

HDC, SKG, MSAG, and MEA conceived of and designed the study. SJK, MSS, CBK, and MEA supervised experimental and statistical analysis. HDC, SKG, MSAG, LCG, and GD performed experiments. DS, CS, GBKH, MB, BFH, TPL, IM, CFVR, MKG, SZP, BDW, GA, DRB, MLR, and MSS provided critical reagents and reviewed data resulting from their use. HDC and MEA wrote the manuscript. All authors critically reviewed the manuscript text.

## Figures and Tables

**Table 2:**
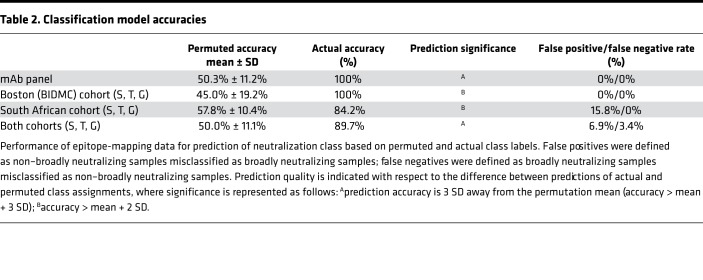
Classification model accuracies

**Table 1:**
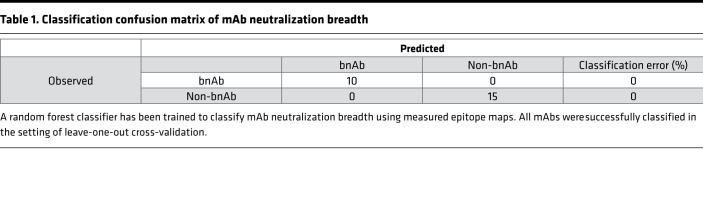
Classification confusion matrix of mAb neutralization breadth

## Version 1 03/08/2018

Electronic publication

## References

[1] Cheung WC (2012). A proteomics approach for the identification and cloning of monoclonal antibodies from serum. Nat Biotechnol.

[2] Reddy ST (2010). Monoclonal antibodies isolated without screening by analyzing the variable-gene repertoire of plasma cells. Nat Biotechnol.

[3] Wu X (2010). Rational design of envelope identifies broadly neutralizing human monoclonal antibodies to HIV-1. Science.

[4] Scheid JF (2011). Sequence and structural convergence of broad and potent HIV antibodies that mimic CD4 binding. Science.

[5] Walker LM (2009). Broad and potent neutralizing antibodies from an African donor reveal a new HIV-1 vaccine target. Science.

[6] Walker LM (2011). Broad neutralization coverage of HIV by multiple highly potent antibodies. Nature.

[7] Bornholdt ZA (2016). Isolation of potent neutralizing antibodies from a survivor of the 2014 Ebola virus outbreak. Science.

[8] Rouvinski A (2015). Recognition determinants of broadly neutralizing human antibodies against dengue viruses. Nature.

[9] Meunier JC (2008). Isolation and characterization of broadly neutralizing human monoclonal antibodies to the e1 glycoprotein of hepatitis C virus. J Virol.

[10] Sui J (2009). Structural and functional bases for broad-spectrum neutralization of avian and human influenza A viruses. Nat Struct Mol Biol.

[11] Tan J (2016). A LAIR1 insertion generates broadly reactive antibodies against malaria variant antigens. Nature.

[12] Shibata R (1999). Neutralizing antibody directed against the HIV-1 envelope glycoprotein can completely block HIV-1/SIV chimeric virus infections of macaque monkeys. Nat Med.

[13] Mascola JR (1999). Protection of Macaques against pathogenic simian/human immunodeficiency virus 89.6PD by passive transfer of neutralizing antibodies. J Virol.

[14] Baba TW (2000). Human neutralizing monoclonal antibodies of the IgG1 subtype protect against mucosal simian-human immunodeficiency virus infection. Nat Med.

[15] Mascola JR (2000). Protection of macaques against vaginal transmission of a pathogenic HIV-1/SIV chimeric virus by passive infusion of neutralizing antibodies. Nat Med.

[16] Parren PW (2001). Antibody protects macaques against vaginal challenge with a pathogenic R5 simian/human immunodeficiency virus at serum levels giving complete neutralization in vitro. J Virol.

[17] Caskey M (2015). Viraemia suppressed in HIV-1-infected humans by broadly neutralizing antibody 3BNC117. Nature.

[18] Scheid JF (2016). HIV-1 antibody 3BNC117 suppresses viral rebound in humans during treatment interruption. Nature.

[19] Schoofs T (2016). HIV-1 therapy with monoclonal antibody 3BNC117 elicits host immune responses against HIV-1. Science.

[20] Bar KJ (2016). Effect of HIV antibody VRC01 on viral rebound after treatment interruption. N Engl J Med.

[21] Lynch RM (2015). Virologic effects of broadly neutralizing antibody VRC01 administration during chronic HIV-1 infection. Sci Transl Med.

[22] Haynes BF (2012). Immune-correlates analysis of an HIV-1 vaccine efficacy trial. N Engl J Med.

[23] Burton DR (1994). Efficient neutralization of primary isolates of HIV-1 by a recombinant human monoclonal antibody. Science.

[24] Huang J (2016). Identification of a CD4-binding-site antibody to HIV that evolved near-pan neutralization breadth. Immunity.

[25] Walker LM (2011). Rapid development of glycan-specific, broad, and potent anti-HIV-1 gp120 neutralizing antibodies in an R5 SIV/HIV chimeric virus infected macaque. Proc Natl Acad Sci USA.

[26] Falkowska E (2014). Broadly neutralizing HIV antibodies define a glycan-dependent epitope on the prefusion conformation of gp41 on cleaved envelope trimers. Immunity.

[27] van Gils MJ (2016). An HIV-1 antibody from an elite neutralizer implicates the fusion peptide as a site of vulnerability. Nat Microbiol.

[28] Lynch RM (2012). The development of CD4 binding site antibodies during HIV-1 infection. J Virol.

[29] Wu X (2011). Focused evolution of HIV-1 neutralizing antibodies revealed by structures and deep sequencing. Science.

[30] Zhou T (2010). Structural basis for broad and potent neutralization of HIV-1 by antibody VRC01. Science.

[31] Zhou T (2007). Structural definition of a conserved neutralization epitope on HIV-1 gp120. Nature.

[32] Sundling C (2012). High-resolution definition of vaccine-elicited B cell responses against the HIV primary receptor binding site. Sci Transl Med.

[33] Thali M (1992). Discontinuous, conserved neutralization epitopes overlapping the CD4-binding region of human immunodeficiency virus type 1 gp120 envelope glycoprotein. J Virol.

[34] Mata-Fink J (2013). Rapid conformational epitope mapping of anti-gp120 antibodies with a designed mutant panel displayed on yeast. J Mol Biol.

[35] Georgiev IS (2013). Delineating antibody recognition in polyclonal sera from patterns of HIV-1 isolate neutralization. Science.

[36] Shmelkov E, Krachmarov C, Grigoryan AV, Pinter A, Statnikov A, Cardozo T (2014). Computational prediction of neutralization epitopes targeted by human anti-V3 HIV monoclonal antibodies. PLoS One.

[37] Ferguson AL, Falkowska E, Walker LM, Seaman MS, Burton DR, Chakraborty AK (2013). Computational prediction of broadly neutralizing HIV-1 antibody epitopes from neutralization activity data. PLoS One.

[38] West AP, Scharf L, Horwitz J, Klein F, Nussenzweig MC, Bjorkman PJ (2013). Computational analysis of anti-HIV-1 antibody neutralization panel data to identify potential functional epitope residues. Proc Natl Acad Sci USA.

[39] Evans MC (2014). Predicting HIV-1 broadly neutralizing antibody epitope networks using neutralization titers and a novel computational method. BMC Bioinformatics.

[40] Doria-Rose NA (2017). Mapping polyclonal HIV-1 antibody responses via next-generation neutralization fingerprinting. PLoS Pathog.

[41] Li L (2015). A broad range of mutations in HIV-1 neutralizing human monoclonal antibodies specific for V2, V3, and the CD4 binding site. Mol Immunol.

[42] Sundling C (2010). Soluble HIV-1 Env trimers in adjuvant elicit potent and diverse functional B cell responses in primates. J Exp Med.

[43] Breiman L (2001). Random Forests. Mach Learn.

[44] Thali M, Olshevsky U, Furman C, Gabuzda D, Li J, Sodroski J (1991). Effects of changes in gp120-CD4 binding affinity on human immunodeficiency virus type 1 envelope glycoprotein function and soluble CD4 sensitivity. J Virol.

[45] Hraber P, Seaman MS, Bailer RT, Mascola JR, Montefiori DC, Korber BT (2014). Prevalence of broadly neutralizing antibody responses during chronic HIV-1 infection. AIDS.

[46] Li Y (2007). Broad HIV-1 neutralization mediated by CD4-binding site antibodies. Nat Med.

[47] Mascola JR, Haynes BF (2013). HIV-1 neutralizing antibodies: understanding nature’s pathways. Immunol Rev.

[48] Ingale J (2014). Hyperglycosylated stable core immunogens designed to present the CD4 binding site are preferentially recognized by broadly neutralizing antibodies. J Virol.

[49] Pejchal R (2011). A potent and broad neutralizing antibody recognizes and penetrates the HIV glycan shield. Science.

[50] Azoitei ML (2011). Computation-guided backbone grafting of a discontinuous motif onto a protein scaffold. Science.

[51] Jardine J (2013). Rational HIV immunogen design to target specific germline B cell receptors. Science.

[52] Bonsignori M (2016). Maturation pathway from germline to broad HIV-1 neutralizer of a CD4-mimic antibody. Cell.

[53] Easterhoff D (2017). Boosting of HIV envelope CD4 binding site antibodies with long variable heavy third complementarity determining region in the randomized double blind RV305 HIV-1 vaccine trial. PLoS Pathog.

[54] Bonsignori M (2014). An autoreactive antibody from an SLE/HIV-1 individual broadly neutralizes HIV-1. J Clin Invest.

[55] Bilska M, Tang H, Montefiori DC (2017). Short communication: Potential risk of replication-competent virus in HIV-1 Env-pseudotyped virus preparations. AIDS Res Hum Retroviruses.

[56] Parren PW (1997). Relevance of the antibody response against human immunodeficiency virus type 1 envelope to vaccine design. Immunol Lett.

[57] Jia M, Lu H, Markowitz M, Cheng-Mayer C, Wu X (2016). Development of broadly neutralizing antibodies and their mapping by monomeric gp120 in human immunodeficiency virus type 1-infected humans and simian-human immunodeficiency virus SHIVSF162P3N-infected macaques. J Virol.

[58] Roben P, Moore JP, Thali M, Sodroski J, Barbas CF, Burton DR (1994). Recognition properties of a panel of human recombinant Fab fragments to the CD4 binding site of gp120 that show differing abilities to neutralize human immunodeficiency virus type 1. J Virol.

[59] von Reyn CF (2010). Prevention of tuberculosis in Bacille Calmette-Guérin-primed, HIV-infected adults boosted with an inactivated whole-cell mycobacterial vaccine. AIDS.

[60] Robb ML (2016). Prospective study of acute HIV-1 infection in adults in East Africa and Thailand. N Engl J Med.

[61] Montefiori DC (2009). Measuring HIV neutralization in a luciferase reporter gene assay. Methods Mol Biol.

[62] Chao G, Lau WL, Hackel BJ, Sazinsky SL, Lippow SM, Wittrup KD (2006). Isolating and engineering human antibodies using yeast surface display. Nat Protoc.

[63] Boesch AW (2014). Highly parallel characterization of IgG Fc binding interactions. MAbs.

[64] Brown EP (2012). High-throughput, multiplexed IgG subclassing of antigen-specific antibodies from clinical samples. J Immunol Methods.

[65] Friedman J, Hastie T, Tibshirani R (2010). Regularization paths for generalized linear models via coordinate descent. J Stat Softw.

[66] Touw WG (2013). Data mining in the Life Sciences with Random Forest: a walk in the park or lost in the jungle?. Brief Bioinformatics.

